# Supplementary material for: Netcombin: An algorithm for constructing optimal phylogenetic network from rooted triplets
Source: PLoS One. 2020 Sep 18;15(9):e0227842. doi: 10.1371/journal.pone.0227842 (PMC7500971; doi:10.1371/journal.pone.0227842)
Supplement: S4 File — (ZIP) [file pone.0227842.s004.zip › SequenceData-Triplet/sequenceData-10-20-30-0-Triplet/length 500, numseq 30, rate 10^-7/1 seq.pdf]

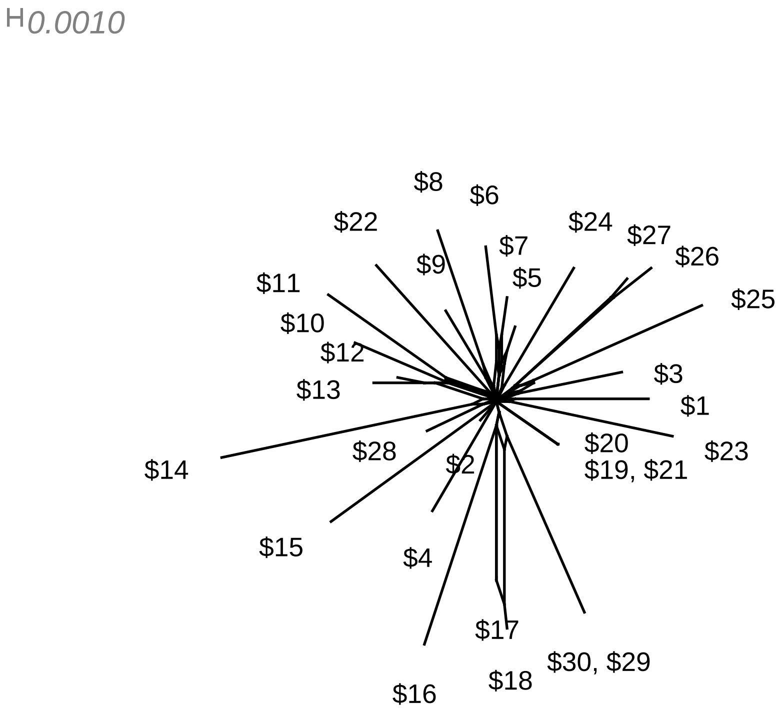

H 0.0010
$8
$6
$22
$24
$7
$27
$26
$9
$5
$11
$25
$10
$12
$3
$13
$1
$20
$23
$28
$14
$2
$19, $21
$15
$4
$17
$30, $29
$18
$16
